# The impact of general self-efficacy and the severity of malocclusion on acceptance of removable orthodontic appliances in 10- to 12-year-old patients

**DOI:** 10.1186/s12903-020-01293-2

**Published:** 2020-11-30

**Authors:** Navid Naseri, Tahereh Baherimoghadam, Niloofar Bassagh, Shahram Hamedani, Elmira Bassagh, Zahra Hashemi

**Affiliations:** 1 Department of Orthodontic, School of Dentistry, Shiraz Branch, Islamic Azad University, Shiraz, Iran; 2 Oral and Dental Disease Research Center, School of Dentistry, Shiraz University of Medical Sciences, Shiraz, Iran; 3 Department of Counseling Center, Shiraz University of Medical Science, Shiraz, Iran; 4 Department of Pediatric Dentistry, School of Dentistry, Yasuj University of Medical Sciences, Yasuj, Iran

**Keywords:** Psychological adjustment, Index of orthodontic treatment need, Acceptance of removable orthodontic appliances, Compliance treatment

## Abstract

**Background:**

The patients’ acceptance of a treatment plan and their subsequent cooperation play a crucial role in achieving the best results in orthodontic treatments. Evidences show some personality traits such as general self-efficacy (GSE) and some dental traits such as severity of malocclusion are correlated with motivation of orthodontic treatment. These factors may predict the patients’ compliance and acceptance in using removable orthodontic appliances. This cross sectional study was conducted to assess the correlation of GSE and the severity of malocclusion with patients’ acceptance in using removable orthodontic appliances.

**Methods:**

This study recruited 50 patients aged 10–12 years who required removable orthodontic appliances. The severity of malocclusion was determined using the index of orthodontic treatment need (IOTN) before the onset of treatment and GSE of participants were assessed using GSE scale self-report. The acceptance questionnaire was proposed to the patients on first (T1), third (T2), and sixth (T3) month after the delivery of the appliance.

**Results:**

The GSE score had a statistically significant correlation with the total score of the acceptance questionnaire, subscale score of satisfaction with the appliance during eating and oral hygiene practice, duration of usage of the appliance, and interest in using it (*P* < 0.05). The IOTN had no significant correlation with the acceptance questionnaire.

**Conclusions:**

Our findings substantiate the role of the GSES, concurrently declining the role of the IOTN in prediction of 10–12-year-old children’s acceptance and cooperation in treatment of malocclusion with removable appliances.

## Background

One of the imperative goals of orthodontic treatments is establishment of a beautiful smile since optimal esthetics is considered as the main reason of referrals in most of patients seeking orthodontic treatments [1, 2]. Removable orthodontic appliances are willingly chosen for treatment of malocclusion in children regarding their low cost and intermittent mode of loading forces [3]. However, these devices have certain drawbacks, which negatively influence patients’ compliance and acceptance that may weaken with time [4–10].

The application of removable appliances in orthodontic treatments necessitates a sufficient level of patient compliance. Evidence shows the cooperation of children who received removable appliances was correlated to their personality traits [11]. Application of General self-efficacy scale (GSES), as well as index of orthodontic treatment need (IOTN) brought the evidence that factors such as patients’ psychological traits and aesthetic demands assessed prior to orthodontic treatment are reliable predictors of future compliance [11, 12]. Such results justify disqualifying selection of removable appliances already at the diagnostic stage.

GSES is a self-report measure of self-efficacy, which is correlated to emotion, optimism. It is the belief in one’s capability to cope with an extensive range of stressful or challenging demands. General self-efficacy (GSE) reflects individual beliefs about how competent they are in performing the behaviors required to bring about the desired outcome [13]. The GSES questionnaire evaluates the confidence of individuals in their capabilities to succeed in different situations. This tool was first designed by Schwarzer and Jerusalem in 1995, and indicates the reliable correlation between the level of healthy behaviors and formation of health-related habits in an individual [13].

IOTN is an objective tool for measurement of the degree and the severity of malocclusion, which subsequently evaluates dental esthetics as well. This index is a beneficial tool that aids professionals in their treatment planning and correction of some dental problems including their unaesthetic appearance [14]. The IOTN has several applications in programming, allocation of resources, and improvement of treatment standards. This index has two components including IOTN- Dental Health Component (DHC) and IOTN-AC (Aesthetic Component); The IOTN-DHC evaluates the severity of malocclusion while the IOTN-AC is used to assess the patient satisfaction with the appearance of the teeth [14].

Studies on the correlation of personality traits with the level of acceptance of orthodontic appliance are limited. The available previous studies on this topic showed that personal attitude towards oral health were an important motivation to seek orthodontic treatment and could affect patients’ tolerance and compliance [15, 16]. However, the question whether the patient’s objectively-verified positive attitude would remain stable and presumably secures adequate cooperation within the course of therapy with removable devices, remains open.

Therefore, we designed our study to evaluate the impact of a personality characteristic (GSE) and severity of malocclusion (IOTN) on acceptance of removable orthodontic appliances in a selected population of Iranian children aged 10–12 years old.

## Methods

### Study design, sample size, and data collection

This study evaluated 58 children aged between 10 and 12 years referred to a private orthodontic office from 1st November to 30th December 2018 for orthodontic treatments in Shiraz/Iran. To determine the sample size, we employed G* power statistical power analysis program 3.1.1 [17]. A sample size of 50 was established, using a power of 0.85 and effect size of *P* = 0.23 for a two-tailed Spearman correlation test (*P* < 0.05). The sample size was increased to 58 to protect the study from any possible future droppings.

The treatment plan of patients included a removable orthodontic appliance with a midline screw along with a labial bow and Adams or Delta clasps on posterior teeth. Moreover, the inclusion criteria were negative history of orthodontic treatment, absence of maxillofacial syndromes such as cleft lip or palate, and absence of any mental disorders. Patients were instructed to use appliance all around the day except during eating meals, drinking hot liquids, and brushing their teeth, and cleaning their appliance.

### Measures

The tools employed in this study were GSES, IOTN-DHC and IOTN-AC, and acceptance of orthodontic appliance questionnaire.

The GSES tool is comprised of 10 items, which are scored according to a 4-point Likert scale. Each item has four answer choices, and the respondents should choose the statement that best describes their condition. The choices include totally opposite to me (score 1), slightly resembles me (score 2), highly resembles me (score 3), and perfectly resembles me (score 4).

This tool is a single-component questionnaire and the scores of the items should be simply summed up to yield the final score. The total score can range from 10 to 40. Higher total scores indicate higher GSE of the individual. In this study, the Persian version of GSES was used. The validity and reliability of the Persian questionnaire have been previously verified [18].

In IOTN-DHC, the severity of malocclusion is classified into five grades. The higher grades indicate greater need for orthodontic treatment. Accordingly, the orthodontic treatment need can be divided into the following groups including group 1 (no need or slight need for orthodontic treatment; Grades 1 and 2), group 2 (moderate need for orthodontic treatment; Grade 3), group 3 (severe need for orthodontic treatment; Grade 4), and group 4 (very severe need for orthodontic treatment; Grade 5).

In IOTN-AC assessment, 10 images of different dentitions are presented to the patients where they are requested to pick the image with the highest resemblance to their dental status. The images are interpreted as: (1) complete satisfaction or slight dissatisfaction with the appearance of the teeth for images 1–4, (2) moderate satisfaction with the appearance of the teeth for images 5–7, and (3) complete dissatisfaction with the appearance of the teeth for images 8–10.

The acceptance questionnaire was used to assess the acceptance of removable orthodontic appliance by patients. It consisted of 10 incomplete statements, which needed to be completed by patient’s choice. The available answer choices were scored using a 6-point Likert scale. In order to help patients understand the answer items, each answer was accompanied by a matching facial expression (Table 1). Scores 5 to 0 were allocated to the answer choices from left to right. The higher scores indicated higher acceptance and satisfaction with the respective item. The total score of this questionnaire ranged from 0 to 55. A higher total score indicated that the problems of using the removable orthodontic appliance were better accepted by patient and reflected privileged motivation to continue the treatment.

**Table 1:**
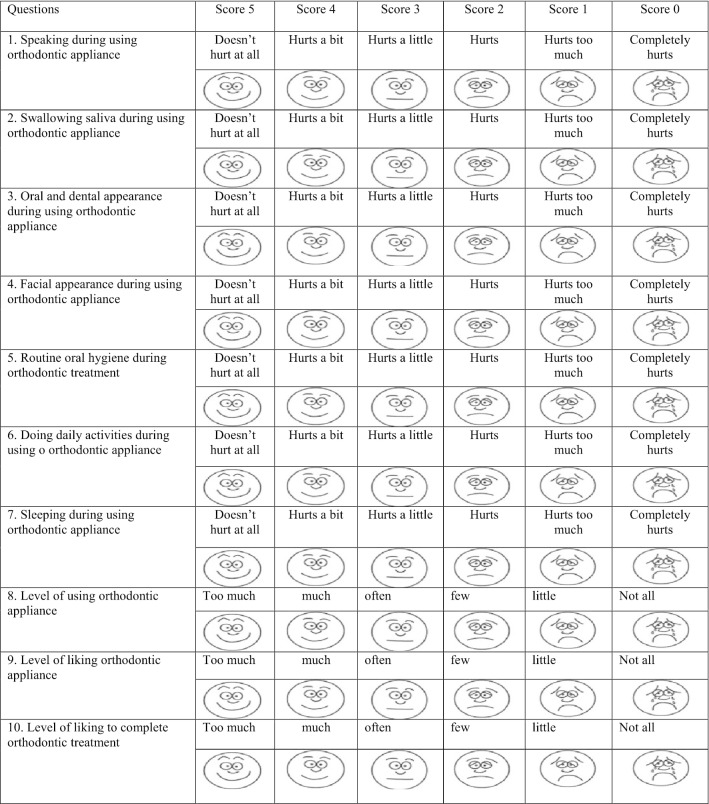
Acceptance of orthodontic appliance scale

After complete elucidation of the nature of the study and all procedures to the participants, the written consent forms were obtained from the parents before the commencement of the study. Afterwards, the participants filled out GSES and IOTN-AC questionnaires; and IOTN-DHC scores were determined for each patient by researcher [T.BM]. Patients filled out these questionnaires in the absence of their parents. The researcher explained the questions in the questionnaires to the patients and instructed them on how to fill it out.

The removable orthodontic appliance was then delivered to the patients and the first follow-up session was scheduled 1 month (T1) after delivery. At this session, the patients filled out the acceptance questionnaire for the first time. The acceptance questionnaire was also proposed to the patients three (T2), and six (T3) months after delivery. The data of all questionnaires were collected and underwent statistical analysis.

### Statistical analysis

Data were analyzed using SPSS version 25 using descriptive and inferential statistics. In inferential statistics, the reliability of Persian version of GSES and acceptance questionnaire were evaluated by Cronbach’s alpha test.

According to the result of one-sample Kolmogorov–Smirnov test for normality assessment of variables, Friedman two-way analysis of variance (ANOVA) and Wilcoxon signed rank test were applied to analyze the changes in individual and total scores yielded from acceptance questionnaire at T1, T2, and T3. The Spearman’s rho was calculated to assess the correlation between the GSES scores and IOTN-DHC, IOTN-AC and mean total and individual scores (for the 10 items) of the acceptance questionnaire filled out at T1, T2, and T3.

## Results

A total of 58 patients were recruited for the study; however, 5 (8.6%) patients filled the questionnaire incompletely and 3 (5.2%) patients did not adhere to the follow up sessions. Hence, these 8 patients were excluded from the study. From 50 patients who completed the study, 21 (42%) were boys (11 ± 1.23 years old) and 29 (58%) were girls (10 ± 0.57 years old).

The Cronbach’s alpha for the Persian version of GSES and the acceptance questionnaire was 0.91 and 0.82 respectively, which indicated good internal consistency of this questionnaire (Table 2).

**Table 2:** Reliability of the acceptance of orthodontic appliance questionnaire

	Item-total correlation	Cronbach’s alpha if item deleted
1. Speaking during using orthodontic appliance	0.296	0.778
2. Swallowing saliva during using orthodontic appliance	0.196	0.794
3. Oral and dental appearance during using orthodontic appliance	0.167	0.784
4. Facial appearance during using orthodontic appliance	0.328	0.774
5. Routine oral hygiene during orthodontic treatment	0.674	0.701
6. Doing daily activities during using orthodontic appliance	0.160	0.794
7. Sleeping during using orthodontic appliance	0.336	0.774
8. Level of using orthodontic appliance	0.676	0.685
9. Level of liking orthodontic appliance	0.163	0.801
10. Level of liking to complete orthodontic treatment	0.485	0.745

Assessment of the normality of variables revealed that data were not normally distributed. Thus, data were analyzed using the non-parametric tests. The results revealed a statistically significant (*P* < 0.05) correlation of GSES and the total score of the acceptance questionnaire (r = 0.486, *P* = 0.001), its subscale regarding oral hygiene (r = 0.303; *P* = 0.032), level of liking orthodontic appliance (r = 0.530, *P* = 0.001) and level of using it (r = 0.296; *P* = 0.037) (Table 3). The IOTN-DHC and IOTN-AC scores had no significant correlation with the mean acceptance questionnaire score of T1, T2, and T3 (Table 4). Comparison of the total and individual scores of the acceptance questionnaire at T1, T2, and T3 revealed no significant change (*P* > 0.05, Table 5).

**Table 3:** The correlation between GSES and acceptance of orthodontic appliance

	Spearman’s correlation	Total score of GSES
1. Speaking during using orthodontic appliance	Correlation coefficient	0.079
	Sig. (2-tailed)	0.587
2. Swallowing saliva during using orthodontic appliance	Correlation coefficient	− 0.081
	Sig. (2-tailed)	0.576
3. Oral and dental appearance during using orthodontic appliance	Correlation coefficient	0.134
	Sig. (2-tailed)	0.352
4. Facial appearance during using orthodontic appliance	Correlation coefficient	0.162
	Sig. (2-tailed)	0.260
5. Routine oral hygiene during orthodontic treatment	Correlation coefficient	0.303
	Sig. (2-tailed)	0.032*
6. Doing daily activities during using orthodontic appliance	Correlation coefficient	0.011
	Sig. (2-tailed)	0.938
7. Sleeping during using orthodontic appliance	Correlation coefficient	0.177
	Sig. (2-tailed)	0.220
8. Level of using orthodontic appliance	Correlation coefficient	0.296
	Sig. (2-tailed)	0.037*
9. Level of liking orthodontic appliance	Correlation coefficient	0.530
	Sig. (2-tailed)	0.000*
10. Level of liking to complete orthodontic treatment	Correlation coefficient	0.109
	Sig. (2-tailed)	0.451
Total score	Correlation coefficient	0.486
	Sig. (2-tailed)	0.000*

*p < 0.05

**Table 4:** The correlation between IOTN components and acceptance of orthodontic appliance

	Spearman correlation	IOTN_AC	IOTN_DHC
1.Speaking during using orthodontic appliance	Correlation coefficient	− 0.001	0.109
	Sig. (2-tailed)	0.994	0.452
2.Swallowing saliva during using orthodontic appliance	Correlation coefficient	0.048	0.139
	Sig. (2-tailed)	0.740	0.336
3. Oral and dental appearance during using orthodontic appliance	Correlation coefficient	− 0.180	0.003
	Sig. (2-tailed)	0.210	0.985
4. Facial appearance during using orthodontic appliance	Correlation coefficient	0.124	− 0.180
	Sig. (2-tailed)	0.393	0.210
5. Routine oral hygiene during orthodontic treatment	Correlation coefficient	− 0.039	0.116
	Sig. (2-tailed)	0.790	0.422
6.Doing daily activities during using orthodontic appliance	Correlation coefficient	− 0.098	− 0.064
	Sig. (2-tailed)	0.498	0.661
7. Sleeping during using orthodontic appliance	Correlation coefficient	0.063	− 0.052
	Sig. (2-tailed)	0.662	0.720
8. Level of using orthodontic appliance	Correlation coefficient	− 0.017	0.082
	Sig. (2-tailed)	0.905	0.573
9. Level of liking orthodontic appliance	Correlation coefficient	0.176	− 0.101
	Sig. (2-tailed)	0.221	0.487
10. Level of liking to complete orthodontic treatment	Correlation coefficient	0.132	− 0.013
	Sig. (2-tailed)	0.360	0.929
Total score	Correlation coefficient	0.057	0.033
	Sig. (2-tailed)	0.694	0.819

**Table 5:** Changes in the mean scores of the acceptance of orthodontic appliance scale within 6 month

	Mean scores of orthodontic appliance acceptance			*P *value
	T1	T2	T3	
1. Speaking during using orthodontic appliance	11.50	12.50	12.04	0.730
2. Swallowing saliva during using orthodontic appliance	6.08	7.79	8.14	0.512
3. Oral and dental appearance during using orthodontic appliance	8.78	8.14	8.13	0.554
4. Facial appearance during using orthodontic appliance	9.79	9.32	10.02	0.419
5. Routine oral hygiene during orthodontic treatment	9.45	10.75	9.45	0.712
6. Doing daily activities during using orthodontic appliance	9.68	7.75	8.14	0.138
7. Sleeping during using orthodontic appliance	7.25	4.50	5.14	0.329
8. Level of using orthodontic appliance	14.90	12.00	13.01	0.502
9. Level of liking orthodontic appliance	14.38	15.77	16.43	0.781
10. Level of liking to complete orthodontic treatment	11.73	12.35	12.15	0.654
Total	22.95	18.67	19.47	0.432

First (T1), third (T2), and sixth (T3) month after the delivery of the appliance

## Discussion

The aim of this study was to define any potential correlation between patients’ acceptance of orthodontic removable appliance during the first 6 months of using this appliance and their GSE, as well as the severity of their malocclusion. For evaluating acceptance level of removable orthodontic appliance, we constructed a 10-item questionnaire. The participants could choose the most appropriate response choice based on their experiences of convenience or discomfort. The assessment of the reliability of this questionnaire was measured with Cronbach’s Alpha and the results revealed an acceptable reliability.

The results of present study indicated moderate positive correlation (r = 0.486, *P* = 0.001) between acceptance of orthodontic appliance and GSES. The results showed that two individual scores of the acceptance questionnaire, i.e. oral hygiene practice in presence of the appliance and level of using appliance had low correlation with GSES; however, the level of liking of appliance showed moderate correlation with GSES. Such results confirmed this assumption that some personality trait of patients may have significant impact on acceptance of orthodontic appliance and patients ‘compliance. Our findings are in line with those of Sarul et al. [11] who found that some personal traits such as GSE might be a valuable tool for foreseeing patients’ cooperation during orthodontic treatment. The findings of our research have verified their results in a different population with different psychological characteristics. In addition; Cooper-Kazaz et al. [19] reported that personality traits and psychological features had significant impact on adjustability and recovery during fixed buccal orthodontic treatment in adult patients. They used Brief Symptom Inventory to assess personality trait. Hansen et al. [20] and Singh et al. [21] observed that personality traits were useful in predicting a patient’s potential willingness and cooperation during fixed orthodontic treatments. The findings of the current study performed on a different population with different psychological and cultural background have confirmed the results yielded by the aforementioned studies.

On the other hand, Amado et al. [22] reported that personality trait of adolescent would not predict the future cooperation and acceptance of appliance during orthodontic treatment. Given a different point of view of cooperation, they reported that personality trait could not be considered as the only factor to predict cooperation, it should be a part of a combination of other factors such as social-economic characteristics, interactions between patient and orthodontist, orthodontist personality and his/her experience [22].

In order to appraise the patients’ acceptance of orthodontic appliances over time, the questionnaire was filled out again 3 and 6 months after delivery. Comparison of the results from the questionnaire filled out before the treatment with these two time episodes revealed that the total and individual scores did not significantly change (*P* > 0.1). In other words, the patients' acceptance remained stable during first 5 months of treatment. This finding is in agreement with the results of Sergl et al. [23] who found no significant change in the frequency of patient complaints regarding orthodontic appliance after 6-month assessment period. Moreover, this result is the evidence-based strengthening point for the research by Sarul et al., who proved that a patient's low evaluation of their smile is a reliable predictor of cooperation, before the beginning of the treatment [24]. Altogether, it allows summing up that use of psychological tests for prediction of a patient’s compliance can be helpful for clinicians who are uncertain whether to start removable orthodontic treatment of malocclusion or to postpone the therapy until camouflage or orthognathic protocols are introduced. Based on a previous study, the idea that patients’ personality trait per se would help predict their compliance and acceptance to a clinically useful degree is no longer plausible [25]. The result of present study might be useful for clinicians, to choose the appropriate patients for being subjected to removable appliance treatments.

In addition to personality trait, in this study we evaluated severity of malocclusion. We employed IOTN-DHC and IOTN-AC to determine malocclusion severity. This index is a highly popular tool that aids clinicians in treatment planning and correction of dental problems such as unaesthetic appearance of the teeth [26]. The result of the present study indicated no significant correlation between acceptance of appliance and IOTN-DHC and IOTN-AC. This finding is inconsistent with the results yielded by Sarul et al. [14], who showed significant correlation between the severity of malocclusion and compliance and cooperation of orthodontic appliance. It has been verified that the more severe the malocclusion the higher patient’s motivation to orthodontic treatment [12, 27]. Previous studies showed that the psychological status in patients with severe malocclusion and scores 4 or 5 of IOTN were significantly affected by malocclusion [28, 29]. The absence of significant correlation between acceptance of appliance and severity of malocclusion can be attributed to low to moderate need for orthodontic treatment among our study population (mean of IOTN-DHC = 2.85 ± 0.56).

### Limitations of the study

Currently there are ways to measure compliance objectively. Tsomos et al. [30] conducted a retrospective cohort study to evaluate the impact of numerous parameters, including sex, age, and prescribed wear time, on compliance. They objectively investigated the compliance of patients who used different types of removable orthodontic appliances in the medium/long period of time. They concluded that objective measures are crucial to evaluate compliance with removable orthodontic appliances since patient compliance is a highly variable subject.

Moreover, the relatively small sample size enrolled in the current study could be considered as a limitation; however, it was impossible for us to increase the sample size since we had to choose patients with similar treatment plan. In addition, we investigated our patient’s acceptance of orthodontic appliance for 6 months; future studies with long term observations would probably bring more consistent results.

## Conclusion

The GSES would be considered as suitable tool for prediction of acceptance of removable orthodontic appliance by 10–12-year-old orthodontic patients; while the severity of malocclusion determined by using the IOTN prior to treatment cannot serve as a good indicator for this purpose. However, the patients’ attitude towards the removable appliance remained unaltered within 6 months from the treatment beginning.

## Data Availability

The datasets used and/or analyzed during the present study are available from the corresponding author.

## Abbreviations

GSES: General self-efficacy scale
GSE: General self-efficacy
IOTN: Index of orthodontic treatment need
DHC: Dental Health Component
AC: Aesthetic Component
